# American Dental Convention

**Published:** 1874-07

**Authors:** J. G. Ambler


### ARTICLE IV.

### *American Dental Convention.*

Mr. Editor—*Dear Sir.*—Permit me, through the medium of your influential journal, to call the attention of its readers to the meeting of the American Dental Convention, which holds its 20th annual session at Saratoga, on the 11th of August.

This organization has done much towards the elevation of our profession. Established upon democratic principles, its doors are open to all reputable practicing dentists.

If a tree is known and appreciated by its fruits, surely this Convention should occupy a prominent position in the dental world, numbering as it does among its members, many of the brightest intellects in our ranks.

The friends of the Convention anticipate a large attendance at its coming session. Dr. T. W. Evans, of Paris, is expected to deliver the opening address, and many of the most prominent and influential members of the profession will be in attendance, read papers and participate in the discussions, &c.

The Transactions for the three past years are now in press, and will be ready for distribution at, or before the time of meeting.

A cordial invitation is extended to every practicing dentist who feels interested in, and desires the advancement of dental science and knowledge, to be present and contribute his mite towards that end, and assist in pushing on the wheels of progress, and thus keep pace with the times and other specialties of Medicine and Surgery.

Let all who love our profession and its advancement be sure to be there.

J. G. AMBLER.

